# Hip-specific and generic patient-reported outcome measure scores after primary hip replacement are associated with early revision surgery: a national registry study

**DOI:** 10.1186/s41687-024-00713-z

**Published:** 2024-03-21

**Authors:** Ilana N. Ackerman, Kara Cashman, Michelle Lorimer, Emma Heath, Ian A. Harris

**Affiliations:** 1 School of Public Health and Preventive Medicine, Monash University, 553 St Kilda Road, Melbourne, VIC 3004 Australia; 2 South Australian Health and Medical Research Institute, North Terrace, Adelaide, South Australia 5000 Australia; 3 School of Clinical Medicine, UNSW Medicine and Health, UNSW Sydney, Sydney, NSW 2052 Australia; 4 Whitlam Orthopaedic Research Centre, Ingham Institute for Applied Medical Research, 1 Campbell Street, Liverpool, NSW 2170 Australia; 5 Australian Orthopaedic Association National Joint Replacement Registry, North Terrace, Adelaide, South Australia 5000 Australia

**Keywords:** Patient-reported outcome measures, Revision hip arthroplasty, Revision hip replacement, Total hip arthroplasty, Total hip replacement

## Abstract

**Background:**

The ability to efficiently identify patients at higher risk of poor outcomes after joint replacement would enable limited resources for post-operative follow-up to be directed to those with the greatest clinical need. This is particularly important as joint replacement rates continue to grow internationally, stretching health system capabilities. Patient-reported outcome measures (PROMs) are routinely administered in many settings and offer an opportunity to detect suboptimal patient outcomes early. This study aimed to determine whether hip-specific and generic PROM scores are associated with early revision hip replacement within six to 24 months after the primary procedure.

**Methods:**

Pre-operative and six-month post-operative PROM scores for patients undergoing primary total hip replacement (THR) were obtained from the Australian Orthopaedic Association National Joint Replacement Registry and Arthroplasty Clinical Outcomes Registry National and linked to revision surgery data. Clinically important improvement was defined using anchor-based thresholds. Associations between PROM scores (hip pain, Oxford Hip Score, HOOS-12, EQ-5D-5L, EQ VAS, patient-perceived change, satisfaction) and revision surgery were evaluated using t-tests, chi-square tests and regression models.

**Results:**

Data were analysed for 21,236 primary THR procedures between 2013 and 2022. Eighty-eight revision procedures were performed at six to 24 months. Patients who were revised had more back pain and worse HOOS-12 scores pre-operatively but between-group differences were small. Worse post-operative PROM scores (hip pain, Oxford, HOOS-12, EQ-5D-5L, EQ VAS) were associated with early revision, after adjusting for age and sex (*p* < 0.001 for all analyses). Patient dissatisfaction (relative risk (RR) 10.18, 95%CI 6.01–17.25) and patient-perceived worsening (RR 19.62, 95%CI 11.33–33.98) were also associated with a higher likelihood of revision. Patients who did not achieve clinically important improvement in hip pain, function, or quality of life had a higher revision risk (RRs 2.54–5.64), compared with those who did (reference).

**Conclusion:**

Six-month hip-specific and generic PROM scores can identify patients at higher risk of early revision surgery. Our data highlight the utility of routine post-operative PROM assessment for signaling suboptimal surgical outcomes.

**Supplementary Information:**

The online version contains supplementary material available at 10.1186/s41687-024-00713-z.

## Background

International arthroplasty registries routinely collect outcomes data on prosthesis failure and revision joint replacement, and numerous registries additionally administer patient-reported outcome measures (PROMs) to provide a comprehensive picture of surgical outcomes [1, 2]. The collection of patient-reported outcomes frequently includes the assessment of pain, function, and quality of life using validated instruments [3]. It is well recognised that PROMs can be used to support clinical care [4, 5]; for example, PROMs can be used to monitor improvements in health outcomes and to communicate patient progress. They may also be valuable for flagging suboptimal patient outcomes after joint replacement, enabling limited resources for post-operative follow-up to be directed to patients with the greatest clinical need [6, 7]. This is particularly pertinent as rates of joint replacement continue to grow internationally [8–10], stretching health system capabilities.

Using national registry data, we have previously shown that worse joint-specific and generic PROMs scores (derived from either single-item or multi-dimensional instruments) at six months after primary total knee replacement were strongly associated with a heightened risk of early revision surgery within two years [11]. Patients who did not achieve thresholds for clinically important improvement in pain, function, or quality of life were most likely to undergo early revision [11], providing practical screening guidance for surgeons [12]. Whether different types of PROMs instruments can similarly identify patients at greater risk of early revision hip replacement is not well understood. Several studies have demonstrated associations between poor PROMs scores and the risk of revision hip replacement, but these have largely focused on hip-specific instruments [6, 13–16] or revision outcomes beyond two years after the primary procedure [6, 15–17]. This study aimed to determine whether hip-specific and generic PROMs scores are associated with early revision hip replacement (defined as revision surgery performed six to 24 months after the primary procedure).

## Methods

### Study design

This study is an analysis of national registry data and is reported according to the REporting of studies Conducted using Observational Routinely-collected Data (RECORD) checklist [18].

### Data sources

The AOANJRR is a national clinical quality registry that collects data on all joint replacements performed in Australia, with well-established data validation procedures [19]. It has captured over 1.85 million joint replacement procedures, with full national coverage since 2003 [19]. The AOANJRR routinely collects data on primary and revision hip replacement (date, side, type of procedure, diagnosis), age, gender, body mass index (BMI) and American Society of Anesthesiologists (ASA) grade. Additionally, pre- and post-operative PROMs data collection has been undertaken by the Arthroplasty Clinical Outcomes Registry National (ACORN) from 2013 to 2018 and by the AOANJRR since 2018. Pre-operative PROMs data were collected within three months prior to surgery and 6-month post-operative data were collected between 5 and 8 months after surgery, to maximise completion rates. ACORN collected PROMs data from patients undergoing primary hip replacement at nine hospitals [20]. The AOANJRR collects PROMs data from patients undergoing primary hip replacement, using methods reported previously [21]. The data used for this study were collected from all 218 hospitals participating in the AOANJRR PROMs program at the time of data analysis (over 300 hospitals contribute data to the AOANJRR but not all hospitals participate in the PROMs program). Person-level linkage of PROMs data to AOANJRR revision surgery data was undertaken through matching patient name, date of birth, operated joint and operated side data. This linkage occurs regularly as part of usual AOANJRR processes. Statisticians at the AOANJRR had full access to all data used for this study.

### Patient-reported outcome measures

Hip-specific and generic PROMs instruments were administered to patients pre- and post-operatively. The instruments administered by the AOANJRR and ACORN at each time point, and completion rates for each instrument, are summarised in the Additional file (Table A1). A hip pain visual analogue scale (VAS) ranging from 0 (no pain) to 10 (worst pain imaginable) was used to assess pain over the previous seven days. A low back pain VAS (0 (no pain) to 10 (worst pain imaginable)) was also administered. The 12-item Oxford Hip Score was used to assess hip-related pain and function (0 (worst) to 48 (best)) [22]. The 12-item HOOS-12 score was administered as an optional measure, given limited evidence of its measurement performance [23, 24]. It provides hip-related pain, function and quality of life domain scores and a summary score (each 0 (worst) to 100 (best)). The EQ-5D-5L instrument was used to evaluate quality of life [25]. An EQ-5D-5L utility score can be generated using country-specific preference weights; utility scores commonly range from less than 0 (indicating quality of life worse than death) to 1.00 (full quality of life). The EQ VAS was used to capture self-reported health (0 (worst health) to 100 (best health)). Three expectation items were also administered pre-operatively for expected hip pain (0 (no pain) to 10 (worst pain)), health (0 (worst health) to 100 (best health), and mobility (5-point scale from ‘no problems’ to ‘severe problems’) in six months’ time. A perceived change question (*How are the problems now with your hip on which you had surgery, compared to before you had your operation?*) and a satisfaction question (*How satisfied are you with the results of your hip replacement?*) were also administered post-operatively, with five response options ranging from ‘much better’ to ‘much worse’ and ‘very dissatisfied’ to ‘very satisfied’, respectively.

#### Study cohort

**Fig. 1:**
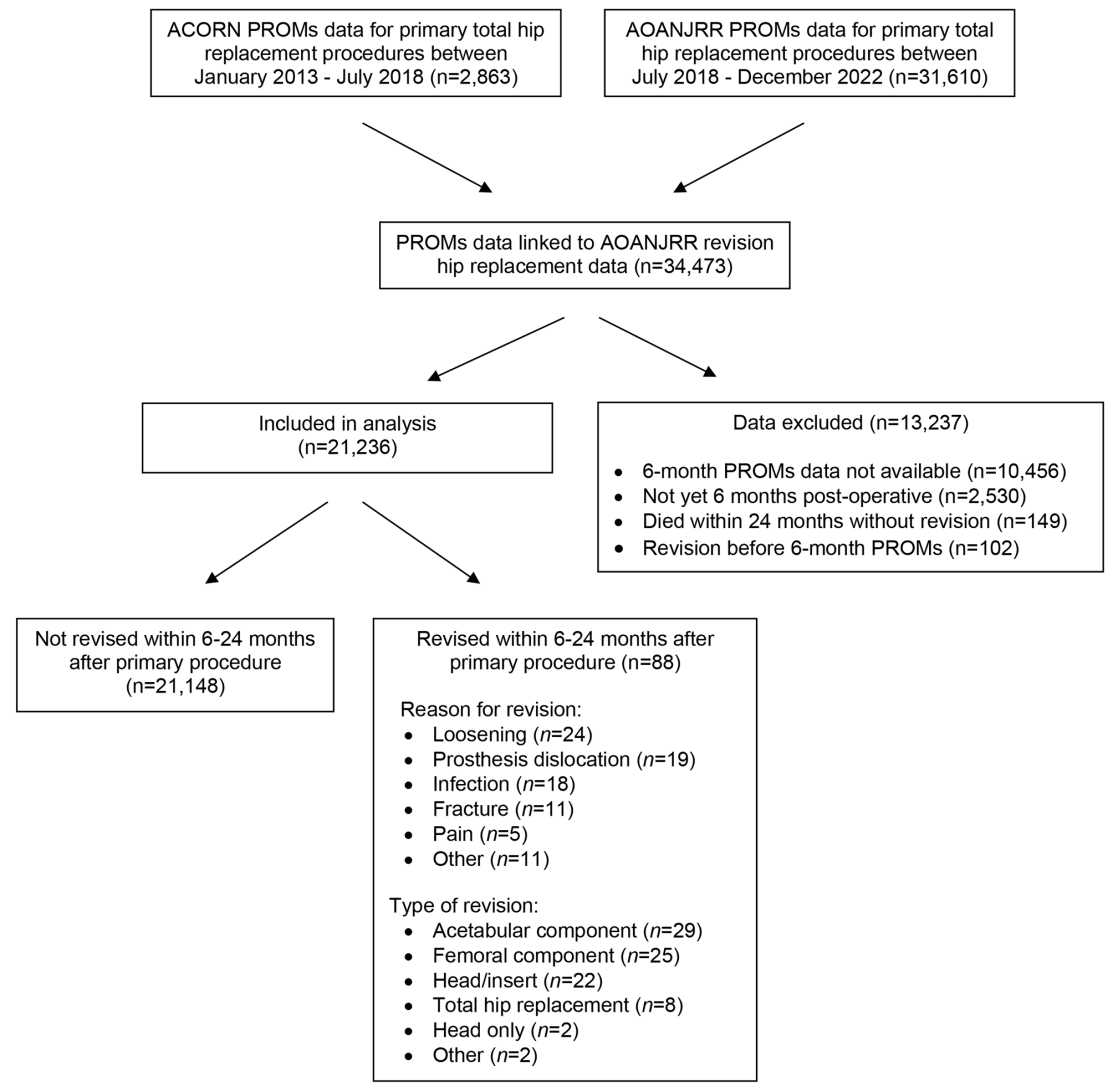
Study cohort

Between January 2013 and December 2022, PROMs data for 34,473 primary THR procedures were available from the ACORN and AOANJRR and linked to AOANJRR data on revision hip replacement (Fig. 1). We considered patients who provided post-operative PROMs data for at least one instrument and either received revision hip replacement (of any type and for any diagnosis) within six to 24 months after the primary procedure or did not receive revision hip replacement but were alive at 24 months or the end of the follow-up period (27 April 2023). Consistent with the methods used previously [11], we excluded those who did not provide post-operative PROMs data, had not yet reached 6 months post-operatively, had died within 24 months without receiving revision hip replacement, or had undergone revision prior to completing post-operative PROMs. The latter group was excluded as they did not reach the post-operative PROMs follow-up point. As shown in Fig. 1, we excluded data for 13,237 primary THR procedures, leaving data from 21,236 procedures for analysis.

### Data analysis

Pre- and post-operative scores for the Oxford Hip Score and HOOS-12 were computed according to published algorithms [23, 26], EQ-5D-5L utility scores were calculated using Australian preference weights [27]. Demographic and clinical data were analysed descriptively. Differences in PROMs scores (pre-operative, post-operative and change scores) between patients who received revision hip replacement and those who did not were evaluated using independent t-tests or chi-square tests, as appropriate. A confidence interval calculator [28] was used to estimate the likelihood of revision for patients who were ‘dissatisfied’ or ‘very dissatisfied’ at six months versus those who were ‘satisfied’ or ‘very satisfied’, and for patients who perceived they were ‘a little worse’ or ‘much worse’ at six months versus those who were ‘a little better’ or ‘much better’. Poisson regression models with robust error variance were used to calculate the relative risk (RR) of revision hip replacement for a one-unit increase in post-operative PROMs score. The models accounted for varying follow-up times [29]. We have used this statistical approach previously for revision knee replacement outcomes [11]. Poisson regression models with robust error variance were also used to evaluate whether clinically important improvement (defined using published anchor-based minimal important change estimates for each PROM instrument: 2 points for hip pain [30, 31], 12.4 points for Oxford Hip Score [32], 19.2 points for HOOS-12 pain [33], 15.7 points for HOOS-12 function [33], 17.2 points for HOOS-12 quality of life [33], 17.9 points for HOOS-12 summary [33], 0.41 utility units for EQ-5D-5L [34], and 9.34 points for EQ-VAS [34]) was associated with early revision. Patients who met the minimal important change threshold were the reference group (relative risk of 1.00). For all PROMs scores, both unadjusted models and models adjusted for age, gender, and pre-operative PROM score were undertaken. Statistical analysis was performed using SAS software version 9.4 (SAS Institute Inc., Cary, North Carolina), with a significance threshold of 0.05.

## Results

### Patients receiving revision surgery

Within the cohort, 88 primary THR procedures were revised within six to 24 months (Fig. 1). The median (IQR) time from primary total hip replacement to revision was 367 (259–556) days and the median (IQR) time from post-operative PROMs completion to revision was 191 (68–383) days. The most common reason for revision hip replacement was loosening (*n* = 24, 27%), followed by prosthesis dislocation (*n* = 19, 22%), infection (*n* = 18, 21%), fracture (*n* = 11, 13%), and pain (*n* = 5, 6%). Revision of the acetabular component was most common (*n* = 29, 33%), followed by revision of the femoral component (*n* = 25, 28%) and head/insert revision (*n* = 22, 25%).

### Pre-operative characteristics and patient-reported outcome measure scores

The demographic and clinical characteristics of patients who underwent revision surgery and those who did not are presented in Table 1. Both groups were similar with respect to average age, proportion of females, average BMI, and ASA grade. Osteoarthritis was the most common primary diagnosis for both groups. Patients in the revised group had more back pain and worse HOOS-12 scores before surgery, compared with the non-revised group (Table 1). However, the between-group differences were small (< 1 point difference in low back pain VAS; 5.8–7.5 point difference in HOOS-12 subscale or summary scores) and unlikely to be clinically important with respect to thresholds for minimal important change. All other pre-operative PROMs scores were comparable between groups.

**Table 1:** Comparison of pre-operative status for primary total hip replacement patients

Variable	Revised at 6–24 months (*n* = 88)	Not revised at 6–24 months (*n* = 21,148)	*p*-value
Demographic and clinical characteristics			
Age in years, mean (SD)	65 (11)	66 (11)	0.50
Body mass index, mean (SD)	31 (6)	30 (7)	0.14
Female, n (%)	45 (51)	11,536 (55)	0.52
ASA grade			0.61
I (Healthy)	7 (8)	1,613 (8)	
II (Mild systemic disease)	44 (50)	11,859 (56)	
III (Severe systemic disease)	35 (40)	7,401 (35)	
IV (Severe systemic disease, threat to life)	2 (2)	204 (1)	
V (Moribund patient)	0 (0)	2 (< 1)	
Primary diagnosis, n (%)			NR
Osteoarthritis	74 (84)	19,712 (93)	
Osteonecrosis	9 (10)	690 (3)	
Other	5 (6)	746 (4)	
Pre-operative scores			
Hip pain VAS, mean (SD)	7.2 (2.0)	6.8 (2.1)	0.13
Low back pain VAS, mean (SD)	5.0 (3.1)	4.2 (3.0)	0.02
Oxford Hip Score, mean (SD)	19.2 (9.5)	20.4 (9.1)	0.23
HOOS-12 Pain, mean (SD)	32.9 (17.3)	38.7 (18.9)	0.05
HOOS-12 Function, mean (SD)	38.5 (20.2)	46.0 (20.4)	0.02
HOOS-12 Quality of life, mean (SD)	24.3 (18.3)	30.8 (19.3)	0.03
HOOS-12 Summary, mean (SD)	31.9 (16.3)	38.5 (17.9)	0.02
EQ-5D-5L utility score, mean (SD)	0.30 (0.40)	0.30 (0.40)	0.18
EQ VAS, mean (SD)	63.9 (21.9)	65.9 (20.4)	0.39
Expected joint pain in 6 months, mean (SD)	2.0 (2.9)	1.6 (2.6)	0.22
Expected health in 6 months, mean (SD)	86.4 (14.7)	87.3 (14.8)	0.63
Expected mobility in 6 months, n (%)			0.55
No problems	49 (74)	11,760 (71)	
Slight problems	11 (17)	3,565 (22)	
Moderate problems	3 (5)	785 (5)	
Severe problems	2 (3)	270 (2)	
Unable to do	1 (2)	80 (1)	

ASA: American Society of Anesthesiologists grade; EQ-5D-5L: Euroqol 5-dimension quality of life index; EQ VAS: Euroqol Health Today Visual Analogue Scale; HOOS-12: 12-item Hip disability and Osteoarthritis Outcome Score; SD: standard deviation; VAS: visual analogue scale

Higher score indicates greater pain (for hip pain VAS and low back pain VAS), better hip-related outcomes (Oxford Hip Score and HOOS-12 scores), higher quality of life (for EQ-5D-5L utility score), better health (for EQ VAS), higher expected joint pain and higher expected health

Numbers may not total *n* = 88 or *n* = 21,148 due to missing data for some variables

NR: *p*-value not reported given the small number of procedures with a primary diagnosis other than osteoarthritis in the ‘revised’ group and unique primary diagnoses in the ‘not revised’ group

### Associations between post-operative patient-reported outcomes and early revision

Table 2 presents the post-operative PROMs scores for patients who received revision surgery and those who did not. Patients who underwent revision demonstrated significantly greater hip pain, greater low back pain, poorer hip-related function and hip-related quality of life, and poorer health and quality of life scores at six months. Effect sizes for the between-group differences ranged from − 1.51 to 0.95. Patients who had early revision demonstrated significantly smaller post-operative improvements in all PROMs scores than those who did not receive revision, with the exception of low back pain for which both groups reported little improvement (Table 2). Apart from low back pain, the magnitude of mean improvement in PROMs scores for the early revision group ranged from 52% to 75% of the mean improvement reported by the non-revised group.

**Table 2:** Comparison of patient-reported outcome scores after primary total hip replacement

Outcome	Revised at 6–24 months (*n* = 88)	Not revised at 6–24 months (*n* = 21,148)	*p*-value	Effect size or relative improvement*
6-month post-operative score, mean (SD)				
Hip pain VAS	3.6 (3.0)	1.5 (2.2)	< 0.01	0.95
Low back pain VAS	3.6 (3.1)	2.8 (2.9)	0.02	0.28
Oxford Hip Score	31.3 (12.6)	41.7 (7.1)	< 0.01	-1.46
HOOS-12 Pain	63.5 (26.5)	87.2 (16.7)	< 0.01	-1.42
HOOS-12 Function	69.4 (24.8)	88.5 (14.2)	< 0.01	-1.35
HOOS-12 Quality of life	53.9 (30.4)	80.6 (19.1)	< 0.01	-1.40
HOOS-12 Summary	62.3 (26.4)	85.4 (15.3)	< 0.01	-1.51
EQ-5D-5L utility score	0.50 (0.40)	0.80 (0.20)	< 0.01	-1.50
EQ VAS	71.4 (18.6)	80.5 (16.0)	< 0.01	-0.57
Baseline to 6-months, mean change (SD)				
Hip pain VAS	-3.5 (3.5)	-5.3 (2.8)	< 0.01	66%
Low back pain VAS	-1.3 (3.5)	-1.4 (3.2)	0.87	93%
Oxford Hip Score	12.3 (13.9)	21.3 (10.1)	< 0.01	58%
HOOS-12 Pain	31.1 (26.5)	47.8 (22.7)	< 0.01	65%
HOOS-12 Function	30.7 (27.1)	41.6 (21.7)	< 0.01	74%
HOOS-12 Quality of life	29.0 (26.1)	49.0 (24.2)	< 0.01	59%
HOOS-12 Summary	30.2 (24.3)	46.1 (20.8)	< 0.01	66%
EQ-5D-5L utility score	0.30 (0.40)	0.40 (0.40)	< 0.01	75%
EQ VAS	7.7 (21.3)	14.7 (21.4)	< 0.01	52%

EQ-5D-5L: Euroqol 5-dimension quality of life index; EQ VAS: Euroqol Health Today Visual Analogue Scale; HOOS-12: 12-item Hip disability and Osteoarthritis Outcome Score; SD: standard deviation; VAS: visual analogue scale

Higher 6-month score indicates greater pain (for hip pain VAS and low back pain VAS), better hip-related outcomes (Oxford Hip Score and HOOS-12 scores), higher quality of life (for EQ-5D-5L utility score) and better health (for EQ VAS)

Positive change scores indicate improvement for the Oxford Hip Score, HOOS-12, EQ-5D-5L and EQ VAS; negative change scores indicate improvement for the hip pain VAS and low back pain VAS

*Effect sizes are presented for the 6-month post-operative scores as an indication of the magnitude of between-group difference; relative improvement is presented for the baseline to 6-month change scores (reported as mean improvement for the revised group, relative to the mean improvement for the non-revised group)

**Fig. 2:**
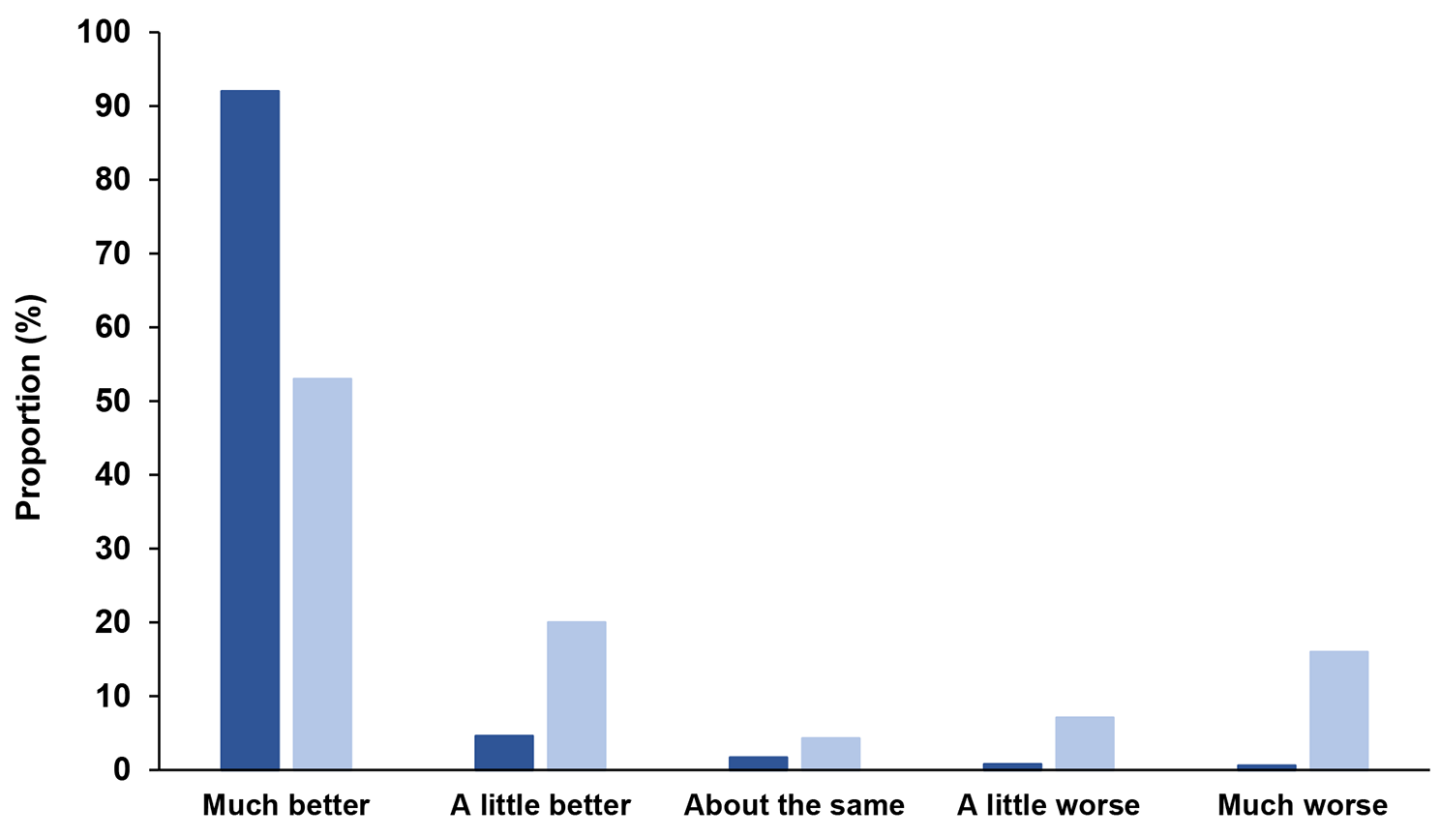
Perceived joint change at six months after primary total hip replacement. Dark blue bars represent the non-revised group and light blue bars represent the revised group. *p* < 0.01 for chi square test

**Fig. 3:**
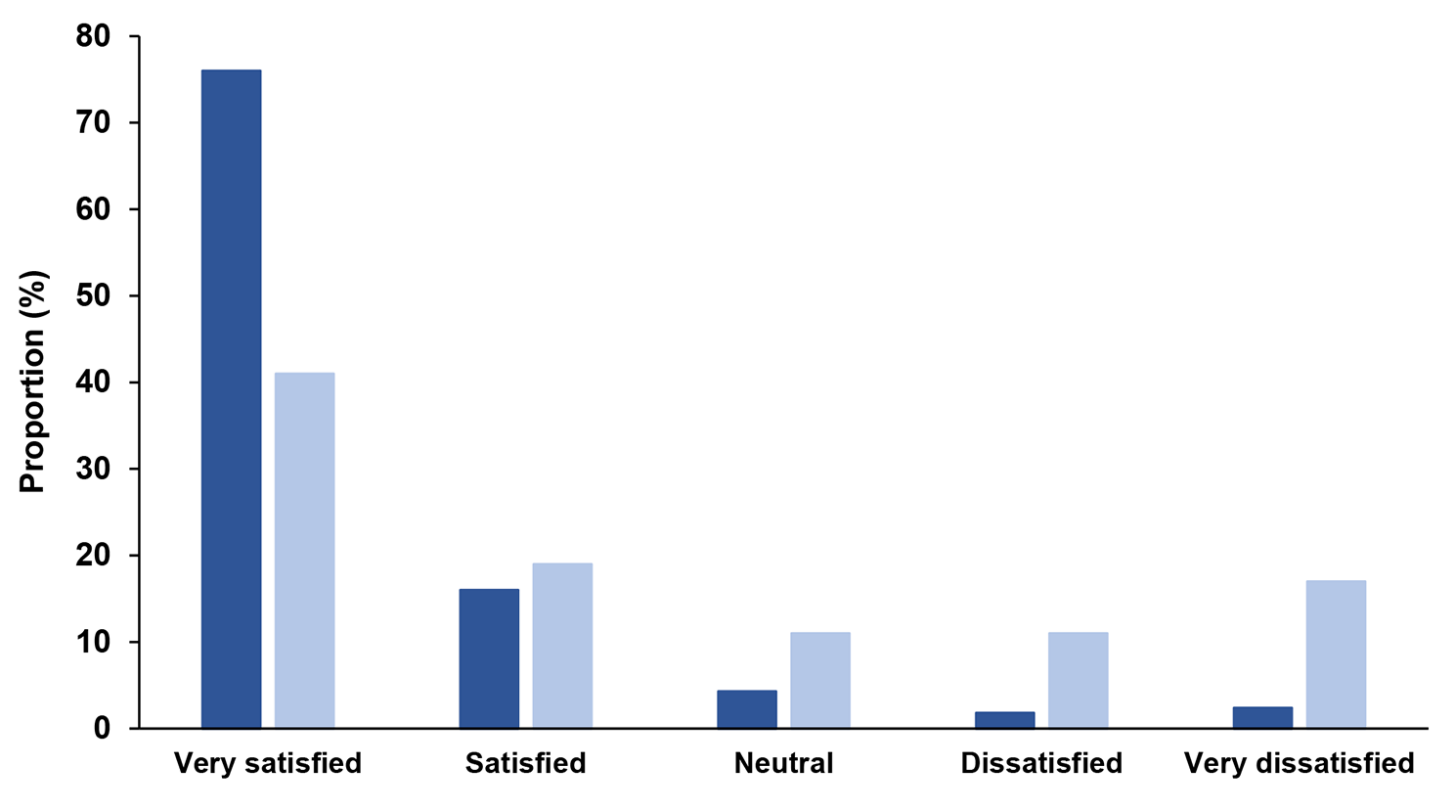
Self-reported satisfaction at six months after primary total hip replacement. Dark blue bars represent the non-revised group and light blue bars represent the revised group. *p* < 0.01 for chi square test

Between-group differences in patient-perceived change were also evident at six months (Fig. 2). Of those revised, 73% perceived their hip was ‘a little better’ or ‘much better’ (versus 97% of the non-revised group) and 23% described their hip as ‘a little worse’ or ‘much worse’ (versus 1% of the non-revised group). Patients who perceived their hip was worse at six months were significantly more likely to undergo early revision than those who perceived their hip was improved (unadjusted RR 19.62, 95%CI 11.33 to 33.98) (Table A3, Additional file). There were also clear differences in post-operative satisfaction. Sixty per cent of patients who received revision were ‘satisfied’ or ‘very satisfied’ with the results of their primary hip replacement (compared to 92% in the non-revised group) and 28% reported they were ‘dissatisfied’ or ‘very dissatisfied’ at this timepoint (Fig. 3). Patients who were dissatisfied at six months were, on average, ten times more likely to undergo early revision (unadjusted RR 10.18, 95%CI 6.01–17.25), compared to those who were satisfied (Table A2, Additional file).

The final regression models included only age and gender as covariates, as the inclusion of variables for which a pre-operative between-group difference was identified (at *p* < 0.05) did not change the results. Each of the post-operative PROMs scores was independently associated with revision hip replacement, with little change in relative risk estimates after adjustment for age and gender (Table 3). As an example, each one-unit increase in hip pain VAS score at six months was associated with a 31% increase in the risk of early revision in the adjusted model (adjusted RR 1.31, 95%CI 1.23 to 1.39). As higher scores represent improvement for the Oxford Hip Score, HOOS-12, EQ-5D-5L and EQ VAS instruments, a one-unit increase in these scores was associated with a significantly reduced risk of early revision (Table 3). For example, a one-unit improvement in the Oxford Hip Score was associated with a 10% reduction in the risk of revision after adjusting for age and gender (adjusted RR 0.90, 95%CI 0.89 to 0.92).

**Table 3:** Associations between post-operative scores and revision hip replacement

Post-operative score	Unadjusted relative risk* (95%CI)	Relative risk adjusted for age and gender** (95%CI)
Hip pain VAS	1.30 (1.23 to 1.38)	1.31 (1.23 to 1.39)
Oxford Hip Score	0.90 (0.89 to 0.92)	0.90 (0.89 to 0.92)
HOOS-12 Pain	0.95 (0.94 to 0.96)	0.95 (0.94 to 0.96)
HOOS-12 Function	0.96 (0.95 to 0.96)	0.96 (0.95 to 0.96)
HOOS-12 Quality of life	0.95 (0.94 to 0.97)	0.95 (0.94 to 0.97)
HOOS-12 Summary	0.95 (0.94 to 0.96)	0.95 (0.94 to 0.96)
EQ-5D-5L utility score	0.12 (0.07 to 0.18)	0.11 (0.07 to 0.18)
EQ VAS	0.98 (0.97 to 0.98)	0.98 (0.97 to 0.98)

*Relative risk of revision hip replacement for a 1-unit increase in PROMs score

**Relative risk of revision hip replacement for a 1-unit increase in PROMs score after adjusting for age and gender

CI: confidence interval; EQ-5D-5L: Euroqol 5-dimension quality of life index; EQ VAS: Euroqol Health Today Visual Analogue Scale; HOOS-12: 12-item Hip disability and Osteoarthritis Outcome Score; VAS: visual analogue scale

Relative risk > 1 indicates a significant increase in likelihood of revision hip replacement; relative risk < 1 indicates significant decrease in likelihood of revision hip replacement

Higher score indicates greater pain (for hip pain VAS), better hip-related outcomes (Oxford Hip Score and HOOS-12 scores), higher quality of life (for EQ-5D-5L utility score) and better health (for EQ VAS)

### Associations between clinically important improvement and early revision

After adjusting for age and gender, patients who did not achieve a clinically important improvement in hip pain had a significantly higher risk of early revision, compared with those who achieved clinically important improvement (adjusted RR 3.95, 95%CI 2.30 to 6.77). A similar pattern was observed for the Oxford Hip Score, HOOS-12 pain, HOOS-12 quality of life, HOOS-12 summary, and EQ-5D-5L scores, as shown in Table 4.

**Table 4:** Clinically important improvement in patient-reported outcomes and early revision

Post-operative variable	Relative risk adjusted for age and gender* (95%CI)
Hip pain VAS	
Achieved MIC (*n* = 14,884)	1.00 (reference)
Did not achieve MIC (*n* = 1,608)	3.95 (2.30 to 6.77)
Oxford Hip Score	
Achieved MIC (*n* = 15,300)	1.00 (reference)
Did not achieve MIC (*n* = 3,570)	5.00 (3.21 to 7.77)
HOOS-12 Pain	
Achieved MIC (*n* = 8,045)	1.00 (reference)
Did not achieve MIC (*n* = 1,221)	5.64 (2.79 to 11.38)
HOOS-12 Function	
Achieved MIC (*n* = 8,193)	1.00 (reference)
Did not achieve MIC (*n* = 1,041)	1.79 (0.73 to 4.39)
HOOS-12 Quality of life	
Achieved MIC (*n* = 8,390)	1.00 (reference)
Did not achieve MIC (*n* = 822)	3.78 (1.75 to 8.14)
HOOS-12 Summary	
Achieved MIC (*n* = 8,377)	1.00 (reference)
Did not achieve MIC (*n* = 835)	3.82 (1.76 to 8.25)
EQ-5D-5L utility score	
Achieved MIC (9,077)	1.00 (reference)
Did not achieve MIC (*n* = 10,019)	2.54 (1.56 to 4.14)
EQ VAS	
Achieved MIC (*n* = 10,922)	1.00 (reference)
Did not achieve MIC (*n* = 7,903)	1.45 (0.93 to 2.23)

EQ-5D-5L: Euroqol 5-dimension quality of life index; EQ VAS: Euroqol Health Today Visual Analogue Scale; HOOS-12: 12-item Hip disability and Osteoarthritis Outcome Score; MIC: minimal important change; VAS: visual analogue scale

*Relative risk > 1 indicates a significant increase in the likelihood of revision hip replacement for patients who did not achieve clinically important improvement, compared to those who did

Thresholds for clinically important improvement were based on the published MIC for each instrument: hip pain VAS (2 points), Oxford Hip Score (12.4 points), HOOS-12 pain (19.2 points), HOOS-12 function (15.7 points), HOOS-12 quality of life (17.2 points), HOOS-12 summary (17.9 points), EQ-5D-5L utility score (0.41 units), and EQ VAS (9.34 points)

## Discussion

Using national registry data, this study provides new evidence that poor hip-specific and generic PROMs scores at six months after primary THR, and smaller post-operative gains in PROMs scores, are associated with a heightened risk of revision surgery within two years. Notably, patients who did not meet thresholds for clinically important improvement in hip pain, hip-related function, hip-related quality of life, or overall quality of life demonstrated a two- to five-fold greater likelihood of early revision. Augmenting our earlier findings in knee replacement [11], these data further emphasise the value of systematically collecting PROMs data before and after joint replacement surgery to flag suboptimal patient outcomes and support clinical care processes.

While early revision was an infrequent outcome in this study (impacting 0.4% of the study cohort), it still represents a considerable burden to patients and the health system at $AUD28,000-$61,000 per revision, depending on procedure complexity [35]. As such, the timely identification of patients most likely to progress to revision surgery is important. Burgeoning rates of elective joint replacement in many countries [8–10] necessitate approaches to post-operative patient follow-up that are less resource-intensive and amenable to large scale-up. The routine use of PROMs instruments to assess patient-centred outcomes (including via remote delivery methods, as used by the AOANJRR) is one such approach and could aid in streamlining clinical follow-up so that limited resources are better targeted to ‘high risk’ patients [6]. We have previously demonstrated that knee-specific and generic PROMs scores at six months after primary total knee replacement can identify patients at greater risk of early revision surgery [11]. In this prior work, patients who did not achieve clinically important improvement were up to eight times more likely (depending on the specific PROM instrument) to undergo revision knee replacement within two years [11]. Our present analysis confirms that six-month hip-specific and generic PROMs scores are similarly informative with respect to detecting likely progression to early revision hip replacement.

This study advances existing knowledge around poor hip-specific PROMs scores and the risk of subsequent revision surgery. Two studies from the New Zealand Joint Registry have reported that worse six-month Oxford Hip Scores were associated with a greater likelihood of revision within two years [13, 14]. Although not adjusted for potential confounders, the analysis undertaken by Rothwell et al. reported a similar association to that observed in the present study; each one-unit decrease in Oxford Hip Score was associated with a 9.7% increase in the risk of revision within two years [13]. One recent study from the AOANJRR reported a weak association between a surgeon’s 2-year cumulative percent revision rate and post-operative Oxford Hip Scores for patients who did not undergo revision, but did not examine revision outcomes at the patient level [36]. In the United States, three studies have examined longer-term PROMs collection and shown that two-year and five-year post-operative hip pain, Mayo Hip Score, and Harris Hip Score (and changes in these scores up to five years after THR) were associated with an increased risk of subsequent THR [6, 15, 16]. In Sweden, hip pain, EQ-5D utility, EQ VAS and satisfaction VAS scores at one year post-operatively were also found to be associated with longer-term revision, up to eight years after THR [17]. In the present study, we applied anchor-based thresholds for improvement, as this is the preferred psychometric approach for determining minimal important change [37]. We are not aware of any other studies that have used similar methods for examining relationships between the magnitude of post-operative improvement and early revision outcomes. Two previous studies used arbitrary cut-off scores to classify improvement in hip-specific PROMs scores at two years. The first study found that patients with either no improvement or worsening in their Mayo Hip Score had a nearly four-fold increase in the likelihood of subsequent revision, compared to patients who reported an improvement of at least 50 points (on a 0–80 scale) [16]. The second study found that patients with either no improvement or worsening in their Harris Hip Score had an 18-fold increase in the risk of subsequent revision, compared to patients who reported improvement of 51–75 points (on a 0-100 scale) [15].

For patients who progressed to early revision in our study, the average time between post-operative PROMs completion and revision surgery was six months. This interval offers time for clinical assessment and potentially, early intervention that could mitigate the need for revision. In our cohort, loosening was the most frequent indication for early revision surgery. Detecting and managing this complication early (given radiographs are not commonly obtained until 12 months post-operatively) may enable patients to avoid a protracted period of pain and impaired function. However, contemporary joint replacement pathways provide little opportunity for clinical review of patients in the first year after joint replacement, and only virtual review clinics in some settings [38, 39]. The collection of six-month PROMs data can provide an early ‘safety net’ for patients whose pain, function and quality of life has not improved as expected or for patients who are dissatisfied with their surgical outcome. Embedding pre- and post-operative PROMs collection within clinical pathways could enable direct contact or expedited review to be initiated, where patients report poor post-operative scores or do not meet thresholds for expected improvement (for example, less than two-point improvement in hip pain VAS [30, 31] or less than 12-point improvement in Oxford Hip Score [32]). This approach is already being used in other clinical specialties, such as oncology care [40]. While we acknowledge that administering multiple PROMs instruments is not feasible in all settings, the single-item measures (joint pain VAS, satisfaction, and perceived change) used in our THR and TKR studies are simple, no-cost, license-free tools that are relatively easy to collect in clinical and registry contexts. Each item was capable of detecting patients at higher risk of early revision hip or knee surgery.

This study had several key strengths, including the use of perioperative PROMs data from a large primary THR cohort that was linked to national data on revision surgery. While earlier studies have focused on hip-specific measures [6, 13–16] or only post-operative PROMs scores [13, 14, 17], we examined a suite of commonly-used hip-specific and generic PROMs instruments and analysed pre-operative, post-operative and change scores with respect to early revision outcomes. We also recognise the study limitations. National arthroplasty registries such as the AOANJRR typically collect a limited set of demographic and clinical data; while the generalisability of the cohort is not known, the age, gender and primary diagnosis characteristics are broadly similar to those reported internationally [41, 42]. The sample size for analysis varied by PROMs instrument, given differences in the AOANJRR and ACORN PROMs programs and some missing data despite direct patient follow-up. We could only include a small number of covariates in the regression models given the number of revision events and we did not adjust for primary diagnosis given the predominance of osteoarthritis. We note the consistent findings across all PROMs instruments with respect to associations with early revision and also the stability of the relative risk estimates in our adjusted models. Together, this suggests that including other variables in the models would likely have little impact. As the AOANJRR PROMs cohort grows over time, opportunities for further multivariate analysis and stratified analysis (for example, based on revision indication) will become increasingly feasible.

## Conclusions

This study demonstrates that both hip-specific and generic PROMs scores offer an opportunity to identify, in a timely manner, patients who are at greater risk of early hip revision. The routine capture of six-month PROMs data provides an efficient mechanism for post-operative patient screening, which can be used to trigger clinical review and implement greater surveillance. Our data indicate that either single-item or multi-item PROM instruments can provide an early signal for a suboptimal surgical outcome.

### Electronic supplementary material

Below is the link to the electronic supplementary material.

Supplementary Material 1

Supplementary Material 2

## Data Availability

Study data are not publicly available under current approvals; however, access to the data analysis code is available upon request.

## Abbreviations

ACORN: Arthroplasty Clinical Outcomes Registry National
AOANJRR: Australian Orthopaedic Association National Joint Replacement Registry
ASA: American Society of Anesthesiologists
BMI: Body mass index
PROM: Patient-reported outcome measure
RR: Relative risk
THR: Total hip replacement
VAS: Visual analogue scale
