# Supplementary material for: Hip-specific and generic patient-reported outcome measure scores after primary hip replacement are associated with early revision surgery: a national registry study
Source: J Patient Rep Outcomes. 2024 Mar 21;8:34. doi: 10.1186/s41687-024-00713-z (PMC10957851; doi:10.1186/s41687-024-00713-z)
Supplement: Supplementary file 1 — Supplementary Material 1 [file 41687_2024_713_MOESM1_ESM.docx]

**Hip-specific and generic patient-reported outcome measure scores after primary hip replacement are associated with early revision surgery:**

**A national registry study**

Ilana N Ackerman, Kara Cashman, Michelle Lorimer, Emma Heath and Ian A Harris

**Additional file**

**Table A1. Available patient-reported outcome measures data**

| **Time point and instrument** | **Data available (n)** | **Proportion of cohort (n=21,236)** |
| --- | --- | --- |
| Pre-operatively |  |  |
| Hip pain VAS* | 16,671 | 79% |
| Low back pain VAS* | 16,766 | 79% |
| Oxford Hip Score** | 19,122 | 90% |
| HOOS-12 pain* | 11,328 | 53% |
| HOOS-12 function* | 11,289 | 53% |
| HOOS-12 quality of life* | 11,265 | 53% |
| HOOS-12 summary* | 11,265 | 53% |
| EQ-5D-5L utility score** | 19,259 | 91% |
| EQ VAS** | 19,114 | 90% |
| Expected joint pain at 6 months* | 16,411 | 77% |
| Expected health at 6 months* | 16,422 | 77% |
| Expected mobility at 6 months* | 16,526 | 78% |
|  |  |  |
| Post-operatively |  |  |
| Hip pain VAS* | 18,241 | 86% |
| Low back pain VAS* | 18,360 | 86% |
| Oxford Hip Score** | 20,895 | 98% |
| HOOS-12 pain* | 12,899 | 61% |
| HOOS-12 function* | 12,885 | 61% |
| HOOS-12 quality of life* | 12,876 | 61% |
| HOOS-12 summary* | 12,876 | 61% |
| EQ-5D-5L utility score** | 21,025 | 99% |
| EQ VAS** | 20,885 | 98% |
| Satisfaction** | 18,232 | 86% |
| Perceived change* | 18,226 | 86% |

EQ-5D-5L: EuroQol five-dimension quality of life index; EQ VAS: Euroqol Health Today Visual Analogue Scale; HOOS-12: 12-item Hip disability and Osteoarthritis Outcome Score

*Instruments collected by the Australian Orthopaedic Association National Joint Replacement Registry Patient-Reported Outcomes Program (2018 onwards); HOOS-12 was administered preoperatively and postoperatively as an optional measure

**Instruments collected by the Arthroplasty Clinical Outcomes Registry National (2013-2018) and the Australian Orthopaedic Association National Joint Replacement Registry Patient-Reported Outcomes Program (2018 onwards)

**Table A2. Joint satisfaction at six months and early revision hip replacement**

|  | **Revised** | **Not revised** | **Total** | **Proportion** |
| --- | --- | --- | --- | --- |
| **Satisfied*** | 42 | 16,630 | 16,672 | 42/16,672 = 0.25% |
| **Dissatisfied**** | 20 | 760 | 780 | 20/780 = 2.56% |

* Patients who reported they were ‘satisfied’ or ‘very satisfied’

** Patients who reported they were ‘dissatisfied’ or ‘very dissatisfied’

**Table A3. Perceived change at six months and early revision hip replacement**

|  | **Revised** | **Not revised** | **Total** | **Proportion** |
| --- | --- | --- | --- | --- |
| **Better*** | 51 | 17,585 | 17,636 | 51/17,636 = 0.29% |
| **Worse**** | 16 | 266 | 282 | 16/282 = 5.67% |

* Patients who reported they were ‘a little better’ or ‘much better’

** Patients who reported they were ‘a little worse’ or ‘much worse’
